# Neuropathological measures of increased tau phosphorylation across the Down syndrome lifespan

**DOI:** 10.1007/s00401-026-02994-8

**Published:** 2026-03-22

**Authors:** Jesse R. Pascual, Isabel Rivera, Halyma Nguyen, Phong T. Ngo, Alan Hoang, Elizabeth J. Andrews, Jeremy Rouanet, Sierra T. Wright, Lorena Sordo, Julia Kofler, Milos D. Ikonomovic, Florence Lai, Mark Mapstone, Bradley T. Christian, Benjamin L. Handen, Ira T. Lott, Eric Doran, Christy L. Hom, Jordan Harp, Frederick Schmitt, Dana L. Tudorascu, Beau M. Ances, Michael Phelan, Lei Liu, Lisi Flores-Aguilar, Elizabeth Head

**Affiliations:** 1 Department of Pathology and Laboratory Medicine, 1111 Gillespie Neuroscience Research Facility, University of California, Irvine, CA 92697 USA; 2 Department of Pathology, University of Pittsburgh School of Medicine, Pittsburgh, PA USA; 3 Department of Neurology, School of Medicine, University of Pittsburgh, Pittsburgh, PA USA; 4 Geriatric Research Education and Clinical Center, Pittsburgh VA Healthcare System, Pittsburgh, PA USA; 5 Department of Psychiatry, University of Pittsburgh School of Medicine, Pittsburgh, PA USA; 6 Department of Neurology, Massachusetts General Hospital, Harvard Medical School, Boston, MA USA; 7 Department of Neurology, University of California, Irvine, Irvine, CA USA; 8 Departments of Medical Physics and Psychiatry, Waisman Center, University of Wisconsin-Madison, Madison, WI USA; 9 Department of Psychiatry, University of Pittsburgh, Pittsburgh, PA USA; 10 Department of Pediatrics, University of California, Irvine, CA USA; 11 Department of Psychiatry and Human Behavior, University of California, Irvine, CA USA; 12 Department of Neurology, University of Kentucky, Lexington, KY USA; 13 Sanders-Brown Center On Aging, University of Kentucky, Lexington, KY USA; 14 Department of Neurology, Washington University School of Medicine, St Louis, MO USA; 15 Brigham and Women’s Hospital, Harvard Medical School, Boston, MA USA

**Keywords:** Alzheimer disease, Trisomy 21, Postmortem, pThr181, pThr217, pThr231

## Abstract

Individuals with Down syndrome (DS) have an increased risk of developing Alzheimer disease (AD), with nearly all individuals exhibiting AD neuropathology, including amyloid beta (Aβ) plaques and neurofibrillary tangles (NFT), by age 40 years. Fluid AD biomarker studies highlight an increase in several phosphorylated tau (p-tau) epitopes in DS. However, neuropathological measures of p-tau epitopes in DS have not been examined. Therefore, our main objective was to characterize p-tau epitope burdens across the DS lifespan at autopsy. We analyzed postmortem brain samples of 98 individuals with late-onset AD (LOAD), DS with AD neuropathology (DSAD), young DS (below 40 years of age), and age-matched neurotypical controls, ranging from 1 to 96 years of age. Immunohistochemical and digital pathology measures of p-tau epitopes at threonine 181 (pThr181), threonine 217 (pThr217), and threonine 231 (pThr231) burdens in the frontal cortex were compared across groups. We observed similar pThr181, pThr217, and pThr231 burdens between DSAD and LOAD, despite DSAD cases being younger on average. Observed pThr181, pThr217, and pThr231 burdens were higher in DSAD compared to young DS and neurotypical controls. Generalized additive models (GAMs) were used to model the cross-sectional trajectory of p-tau epitope burdens across the DS lifespan. Estimated age breakpoints revealed a significant rise in frontal cortex pThr231 at age 40, followed by pThr181 and pThr217 at age 42. In summary, our findings revealed an age-associated increase in p-tau epitopes across the DS lifespan. Our results have the potential to inform future associations between neuropathological and biofluid and neuroimaging biomarker measures of p-tau epitopes.

**Supplementary Information:**

The online version contains supplementary material available at 10.1007/s00401-026-02994-8.

## Introduction

People with Down syndrome (DS) are at high risk of developing Alzheimer disease (AD) due to the triplication of chromosome 21 and related overexpression of the amyloid precursor protein gene (*APP*) [11, 50]. Autopsy studies indicate that significant amyloid beta (Aβ) accumulation in DS begins after 30 years of age [38, 39]. Further, positron emission tomography (PET) imaging in DS suggests that Aβ accumulation begins to rise in the mid-30s and progressively accumulates over time [17, 19, 33, 34]. Neurofibrillary tangles (NFT) composed of hyperphosphorylated tau (p-tau) are typically observed later in DS [16, 19, 47, 58, 63, 65] and their expansion follows a similar Braak staging pattern as reported in late-onset AD (LOAD) [6–8, 10, 64]. Interestingly, neuroimaging studies suggest that the gap in time between the accumulation of Aβ and tau pathology in DS is under five years, which is more rapid than that observed in LOAD (typically ~ 5–9 years) [30, 63]. By the time people with DS reach 40 years of age, virtually all have Aβ and p-tau neuropathology, which is followed by increasing risk for mild cognitive impairment by age 54 [25, 32].

Aβ and p-tau protein levels in cerebrospinal fluid (CSF) and in plasma are increasingly recognized as biomarkers for the early detection of AD, both in the neurotypical population [18, 66] and in DS [12–14, 24, 26, 28, 29, 41, 54, 55]. Increasing biofluid (CSF and plasma) p-tau biomarkers, including pThr181 and pThr217, are recognized for their ability to identify amyloid and tau pathologies in DS [26, 35, 49]. Another common p-tau biomarker, plasma pThr231, along with pThr181, increases around age 39 years in DS, aligning with age trajectories reported in autosomal dominant forms of AD (ADAD) [14, 23, 45]. Neuropathological characterization of p-tau epitopes currently applied in CSF and plasma biomarker assays can provide a better understanding of how tau pathology changes in brain tissue and in biofluids with age and relate to each other in DS. Thus, our goals were to quantify the neuropathology burdens of pThr181, pThr231, and pThr217 in the frontal cortex of autopsy cases with LOAD compared to DS with AD neuropathology (DSAD), and across the DS lifespan. Specifically, we hypothesized that: 1) LOAD and DSAD would show similar burdens for each p-tau protein level; 2) p-tau burdens would be higher in DSAD compared to young DS (herein referred to as DS) and similarly aged control cases; and 3) there would be a differential age-associated increase in all three p-tau epitopes in DS across the lifespan. To test these hypotheses, we used immunohistochemical and digital pathology techniques.

## Methods

### Brain tissue

Postmortem human frontal cortical tissue (Brodmann area 9, BA9) was obtained from the following tissue repositories: the University of California, Irvine Alzheimer Disease Research Center (UCI-ADRC), the University of Kentucky (UKY-ADRC), and the National Institute of Child Health & Development (NICHD) Brain and Tissue Bank for Developmental Disorders at the University of Maryland, Baltimore, MD. The selected autopsy cases ranged from 1 to 96 years of age and were categorized into five groups: late-onset AD (LOAD) with AD neuropathology (*n* = 18, ages 66–96), DS with AD neuropathology (DSAD) (*n* = 32, ages 42–68), young DS (DS) (*n* = 11, ages 1–40), young neurotypical controls (YC) (*n* = 15, ages 1–39), and middle-aged neurotypical controls (MC) (*n* = 22, ages 40–69). The demographics, including age, postmortem interval (PMI), sex, *ApoE4* genotype, and Braak neurofibrillary tangle (NFT) stage, are presented in Table 1. DS cases with mosaicism and partial trisomy were excluded. The groups included in this study were balanced for sex, age, PMI, and presence or absence of AD neuropathology to the extent permitted by available neuropathological data. *APOE* genotype and Braak NFT staging were not available for the YC, MC, and DS groups collected from the NIH Neurobiobank.

**Table 1:**
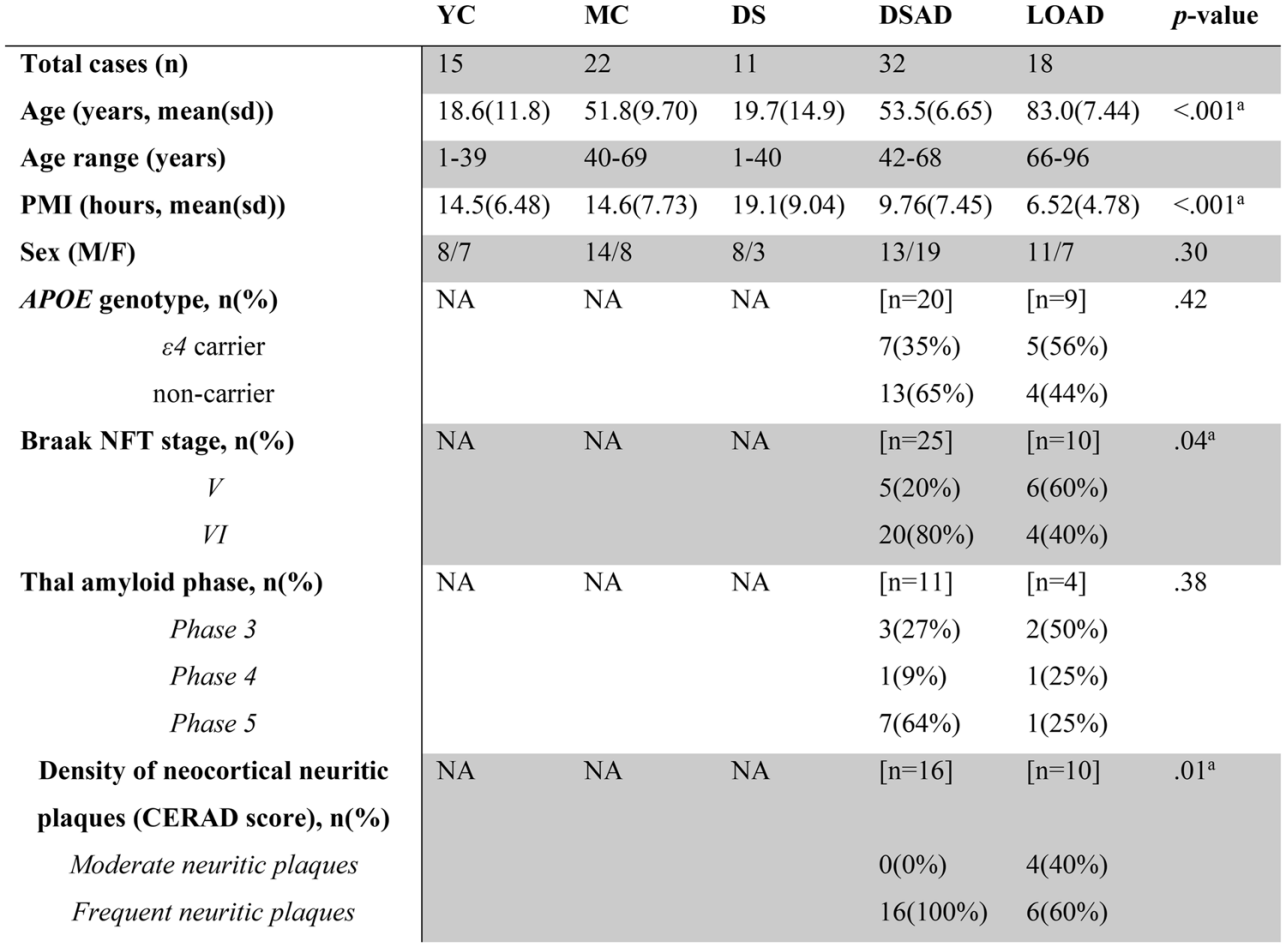
Demographics of postmortem cases

*APOE* ε4 status, Braak NFT stage, Thal amyloid phase, and density of neocortical neuritic plaques (CERAD score) were not available for YC, MC, DS, and a subset of DSAD and LOAD cases. *YC* young controls; *MC* middle age controls; *DS* young Down syndrome; *DSAD* DS with AD neuropathology; *NA* not available; *PMI* postmortem interval; *NFT* neurofibrillary tangle

^a^Significant effects are based on Welch’s ANOVA or Fischer exact tests

### Immunohistochemistry (IHC)

Postmortem fixed (formalin or 4% paraformaldehyde) frontal cortex tissue samples were sectioned serially at 30 µm with a vibratome (Leica Biosystems, Buffalo Grove, Illinois, USA) and stored in phosphate-buffered saline (PBS) with 0.02% sodium azide at 4 °C until used (Suppl. Fig. S1). To ensure a robust and reproducible design, cases from five groups were randomized to experiments and coded to maintain blinding with respect to group. Each experiment was balanced for all diagnostic groups. One or two cases were repeated randomly within an experiment as an internal control. IHC was performed on free-floating tissue sections. Tissue sections were treated with 3% H_2_O_2_/10% methanol and washed in a series of buffers in Tris-buffered saline (TBS), both without and then with Triton X-100. No antigen retrieval was used. Tissue samples were placed in blocking buffer for 1 h at room temperature with TBS, 0.1% Triton, and 5% bovine serum albumin (BSA). Sections were incubated overnight in primary antibodies at 4℃ at low speed on an orbital shaker. The following primary antibodies were used: pThr181 (AT270, mouse monoclonal, 1:8000, Invitrogen), pThr217 (44–744, rabbit polyclonal, 1:8000, Invitrogen), and pThr231 (1H6L6, rabbit monoclonal, 1:8000, Invitrogen) (Table 2). The tissue sections were incubated in the respective biotinylated anti-rabbit or anti-mouse secondary antibodies (Vector Laboratories). An avidin–biotin complex peroxidase kit (VECTASTAIN Elite ABC-HRP Kit) and a 3,3'-diaminobenzidine substrate kit (Vector Laboratories) were used for signal amplification and visualization. Tissue sections were counterstained with cresyl violet, dehydrated in a series of ascending alcohols, cleared with xylene, and coverslipped with Depex mounting media.

**Table 2:**
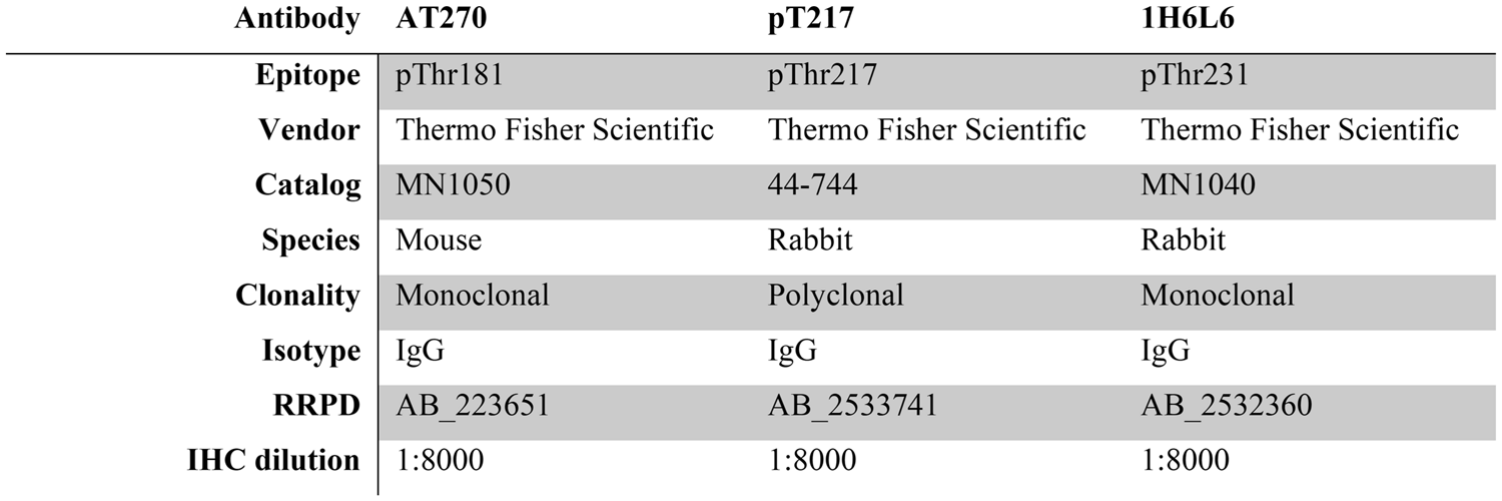
Primary antibodies used for IHC

### Image acquisition

Slides were scanned at 40X magnification using the Leica Aperio Versa 200 and analyzed with Aperio ImageScope Software (v. 12.4.6.5003) using the Positive Pixel Count V9 algorithm, which identified strong and positive pixels (Suppl. Fig. S2). For this, we used a modified approach described by [4]. Briefly, five annotation boxes (600 × 600 × 600 µm) were visually positioned in the gray matter of the frontal cortex. The first box captured an area of highest p-tau pathology, the second box captured an area of least p-tau pathology, and three boxes were randomly selected as described by [44]. Further, this approach ensures reproducibility for future studies. The raw p-tau positive pixels were divided by the total area to calculate the % area covered by p-tau immunoreactivity, which we defined as “burden”. The average of all five regions was calculated to determine the total % burden of p-tau. The p-tau epitope labeling consisted of total tau pathology burden including pretangles, neurofibrillary tangles, neuropil threads, and tau-positive dystrophic neurites [60, 61].

### Statistics

All statistical analyses were performed with R software, version 4.4.1 and 4.4.3. The normality of data was assessed using the Shapiro–Wilk method, followed by nonparametric analysis. To compare demographic characteristics across groups, Welch’s ANOVA was used for age and PMI and Fischer’s exact test was used for sex. Exploratory statistical analysis for *APOE* ε4 status, Braak NFT stage, Thal amyloid phase, and density of neocortical neuritic plaques (CERAD score) between DSAD and LOAD cases were performed using Fischer’s exact test. Spearman rank correlation was used to examine the relationship between PMI and p-tau epitope burdens, stratified by diagnostic group. To compare p-tau epitope burdens between DSAD and LOAD, the Mann–Whitney U test was performed. Partial correlation analysis between pThr181, pThr217, and pThr231 in DSAD and LOAD groups were assessed using Spearman’s method and adjusted for age using the R package “ppcor” [31]. The Kruskal–Wallis test, followed by Dunn’s post hoc analysis with Bonferroni correction, was used to examine p-tau epitope burdens across DSAD, DS, MC, and YC groups. Statistical significance was set at *p* < 0.05. Generalized additive models (GAMs) using nonparametric smoothers were fitted to DS and DSAD cross-sectional data to model the association between p-tau epitope burdens and age. GAMs were assessed using the built-in function in the mgcv package for basis dimension adequacy (*k* = 20) and to determine model fit [62]. The k-index values were approximately 1 (*p* > 0.05), indicating no evidence of underfitting or overfitting. Due to the nonlinear relationship, the Pettitt test was applied to estimate the age (breakpoint) where p-tau epitope burdens significantly changed across the DS lifespan.

## Results

Three p-tau epitopes (pThr181, pThr 217, and pThr 231) were analyzed in 98 frontal cortices, with 44 being female (44.9%), and a mean PMI ranging from 6.5 to 19 h (Table 1). The mean age was 18.6 (SD = 11.8) years for YC, 51.8 (SD = 9.70) years for MC, and 19.7 (SD = 14.9) years for DS. Moreover, the mean age was lower in DSAD [53.5 (SD = 6.65) years] compared with LOAD [83.0 (SD = 7.44) years] cases. *APOE* ε4 status, Braak NFT stage, Thal amyloid phase, and density of neocortical neuritic plaques (CERAD score) were available for a subset of DSAD and LOAD cases. Exploratory analysis shows the DSAD cases trending toward a higher Braak NFT stage (*p* = 0.04) and CERAD neuritic plaques (*p* = 0.01) compared to LOAD. No significant differences were observed for *APOE* ε4 status (*p* = 0.42) and Thal amyloid phase (*p* = 0.38) between DSAD and LOAD groups. The overall distribution of race/ethnicity represented approximately 66% White, 9% African American, 1% Asian, and 23% unknown. Although PMI varied across groups, this was primarily driven by young cases, who had longer PMIs and lower p-tau burden (Suppl. Fig. S3) from the NIH Neurobiobank. In subsequent analyses, we did not covary for PMI.

### P-tau burden is similar in DSAD and LOAD

**Fig. 1:**
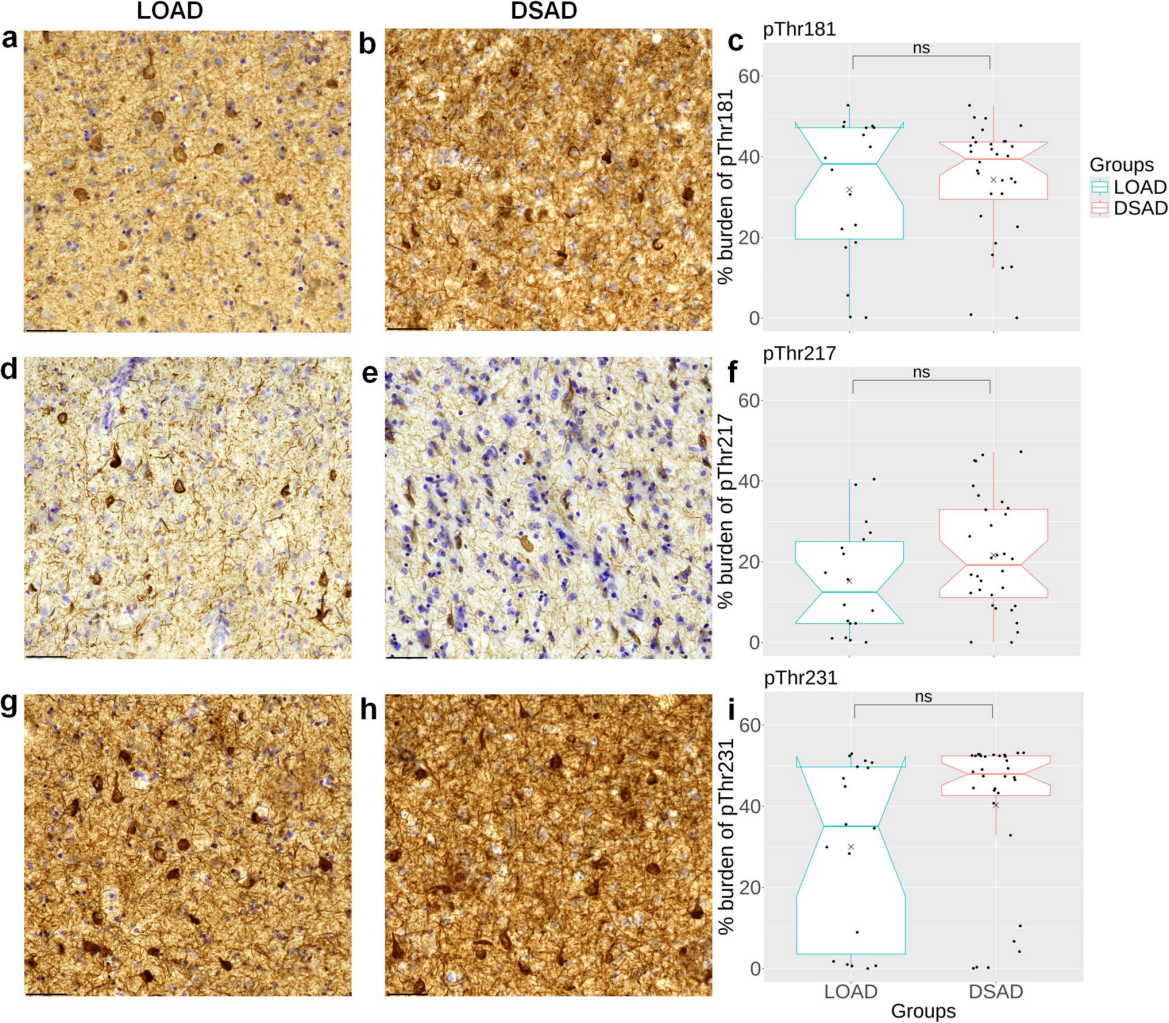
Comparisons of p-tau epitope burdens in DSAD and LOAD. Representative images of **a, b** pThr181, **d, e** pThr217, and **g, h** pThr231 immunohistochemical labeling in the frontal cortex of LOAD and DSAD. Notched box plots show the percentage burden of **c** pThr181, **f** pThr217, and **i** pThr231 across the groups. Dots represent the number of individuals per group: LOAD (*n* = 18) and DSAD (*n* = 32). Statistical comparisons between LOAD and DSAD were performed using the Mann–Whitney *U* test. *LOAD* Late-onset AD; *DSAD* DS with AD neuropathology; *ns* not statistically significant. Scale bar = 50 μm

We first compared p-tau burdens for each epitope between LOAD and DSAD to test the hypothesis that p-tau epitope burdens would be equivalent between the two groups, despite DSAD cases being younger on average. When comparing LOAD to DSAD, there were no significant differences in burden for pThr181 (Fig. 1a–c), pThr217 (Fig. 1d–f), or pThr231 (Fig. 1g–i). For pThr231 pathology, we observed that the DSAD group separated into two clusters, with most cases having higher pThr231 burden (*n* = 26) and a smaller cluster with low pThr231 burden (*n* = 6) (Fig. 1i). Analysis between these subclusters showed no significant differences in age (*p* = 0.68), PMI (*p* = 0.87), or sex (χ^2^(1) = 0.96, *p* = 0.327) (Suppl. Fig. S4). Next, we assessed the association between pThr181, pThr217, and pThr231 in DSAD and LOAD using the Spearman partial correlation while adjusting for age. Associations between pThr181, pThr217, and pThr231 within the DSAD and LOAD groups were positively correlated, with all associations reaching statistical significance (Table S1). Overall, these findings confirm that pThr181, pThr217, and pThr231 pathologies in the frontal cortex in DSAD are similar to LOAD.

### P-tau is elevated in DSAD compared to DS and neurotypical controls

**Fig. 2:**
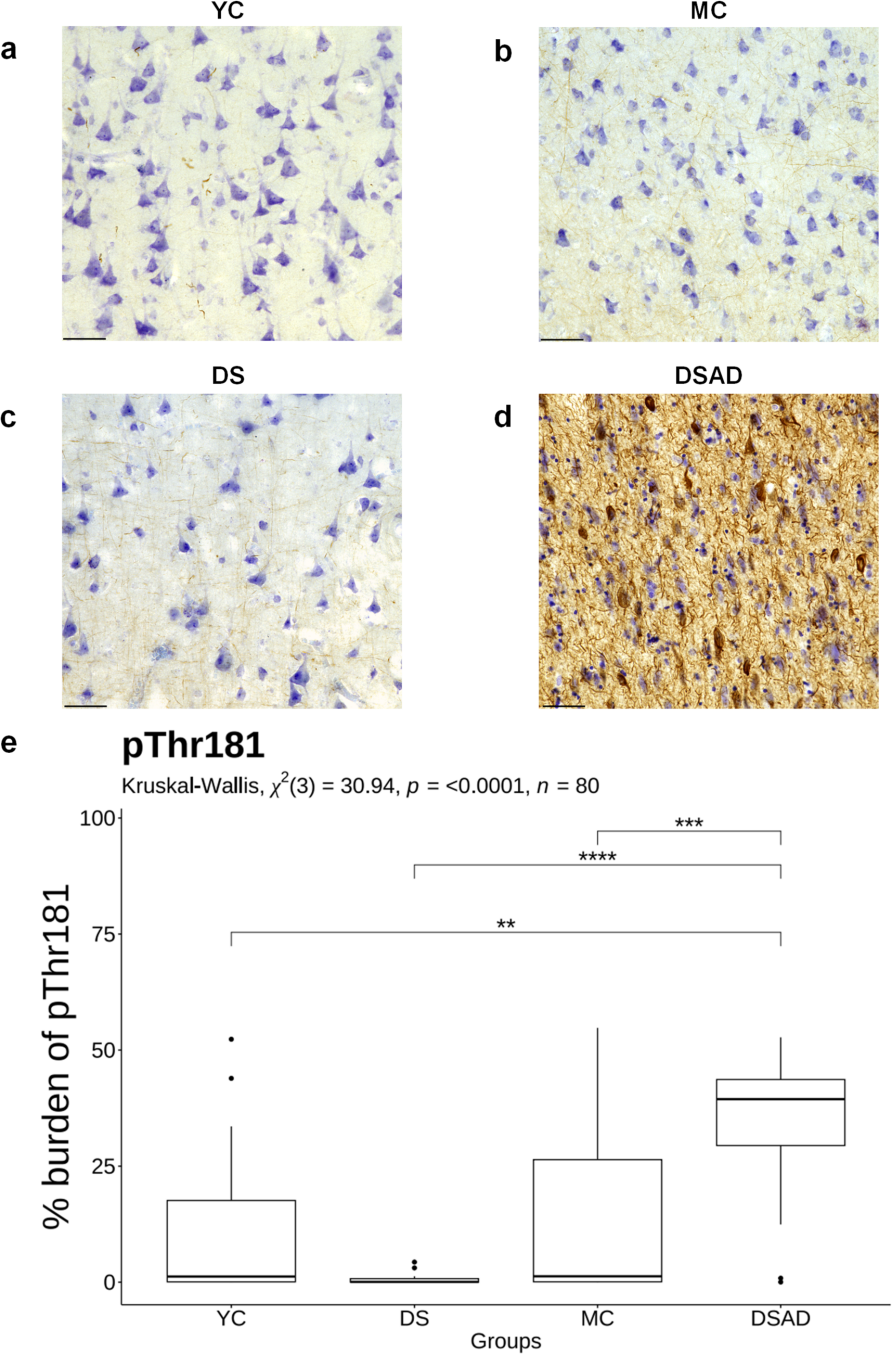
Comparison of pThr181 burden in DSAD, DS, and age-matched controls. Representative images of pThr181 immunohistochemical labeling in the frontal cortex of **a** YC, **b** MC, **c** DS, and **d** DSAD. **e** The box plot shows the percentage burden of pThr181 across the groups. Sample sizes per group are as follows: YC (*n* = 15), MC (*n* = 22), DS (*n* = 11), and DSAD (*n* = 32). Statistical comparisons between groups were performed using the Kruskal–Wallis test, followed by Dunn’s post hoc test with Bonferroni correction. *p < 0.05, **p < 0.01, ***p < 0.001, ****p < 0.0001. *YC* young controls; *MC* middle-aged controls; *DS* young Down syndrome; *DSAD* DS with AD neuropathology; Scale bar = 50 μm

**Fig. 3:**
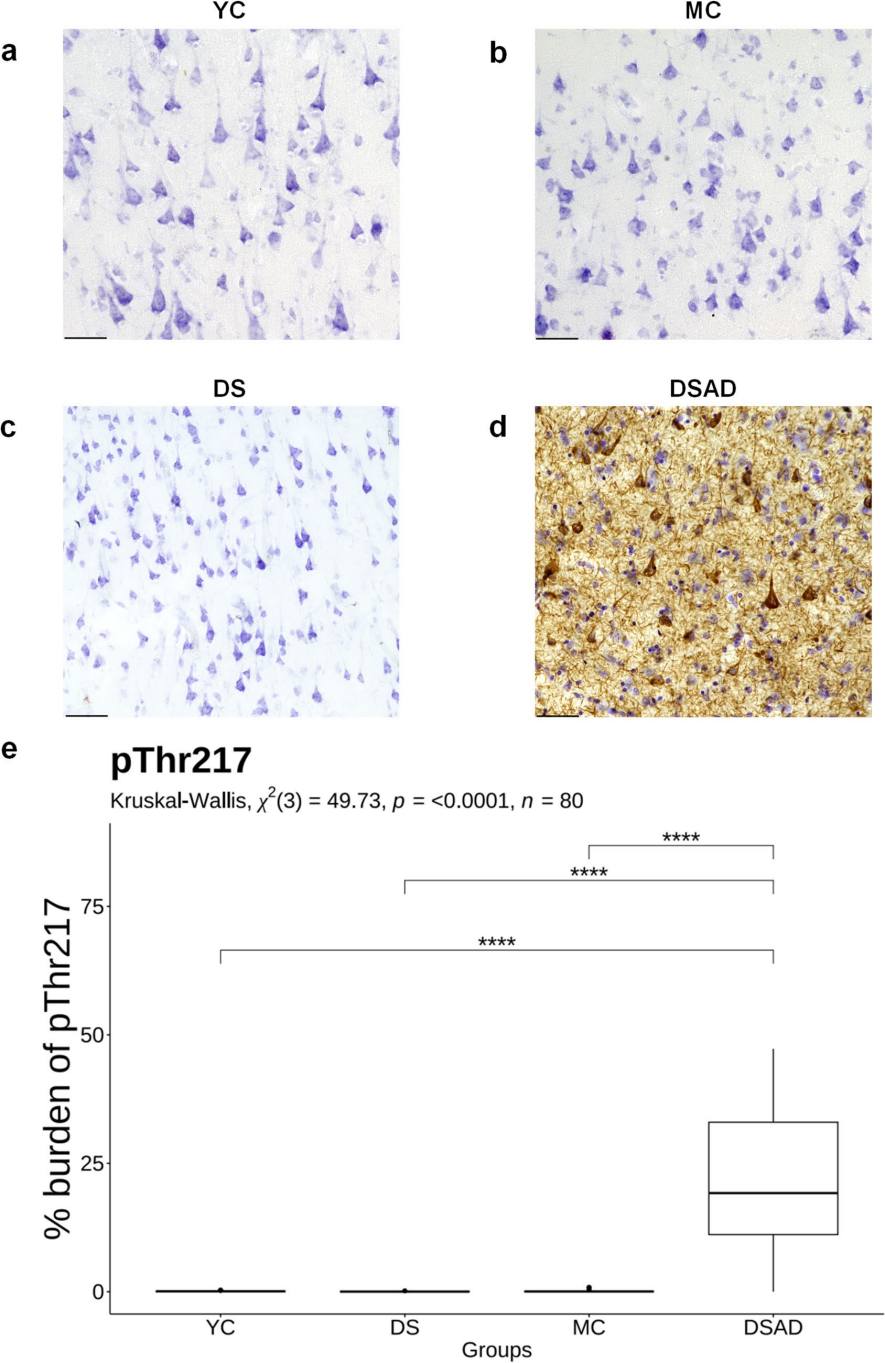
Comparison of pThr217 burden in DSAD and age-matched controls. Representative images of pThr217 immunohistochemical labeling in the frontal cortex of **a** YC, **b** MC, **c** DS, and **d** DSAD. **e** The box plot shows the percentage burden of pThr217 across the groups. Sample sizes per group are as follows: YC (*n* = 15), MC (*n* = 22), DS (*n* = 11), and DSAD (*n* = 32). Statistical comparisons between groups were performed using the Kruskal–Wallis test, followed by Dunn’s post-hoc test with Bonferroni correction. *p < 0.05, **p < 0.01, ***p < 0.001, ****p < 0.0001. *YC* young controls; *MC* middle-aged controls; *DS* young Down syndrome; *DSAD* DS with AD neuropathology; Scale bar=50 μm

**Fig. 4:**
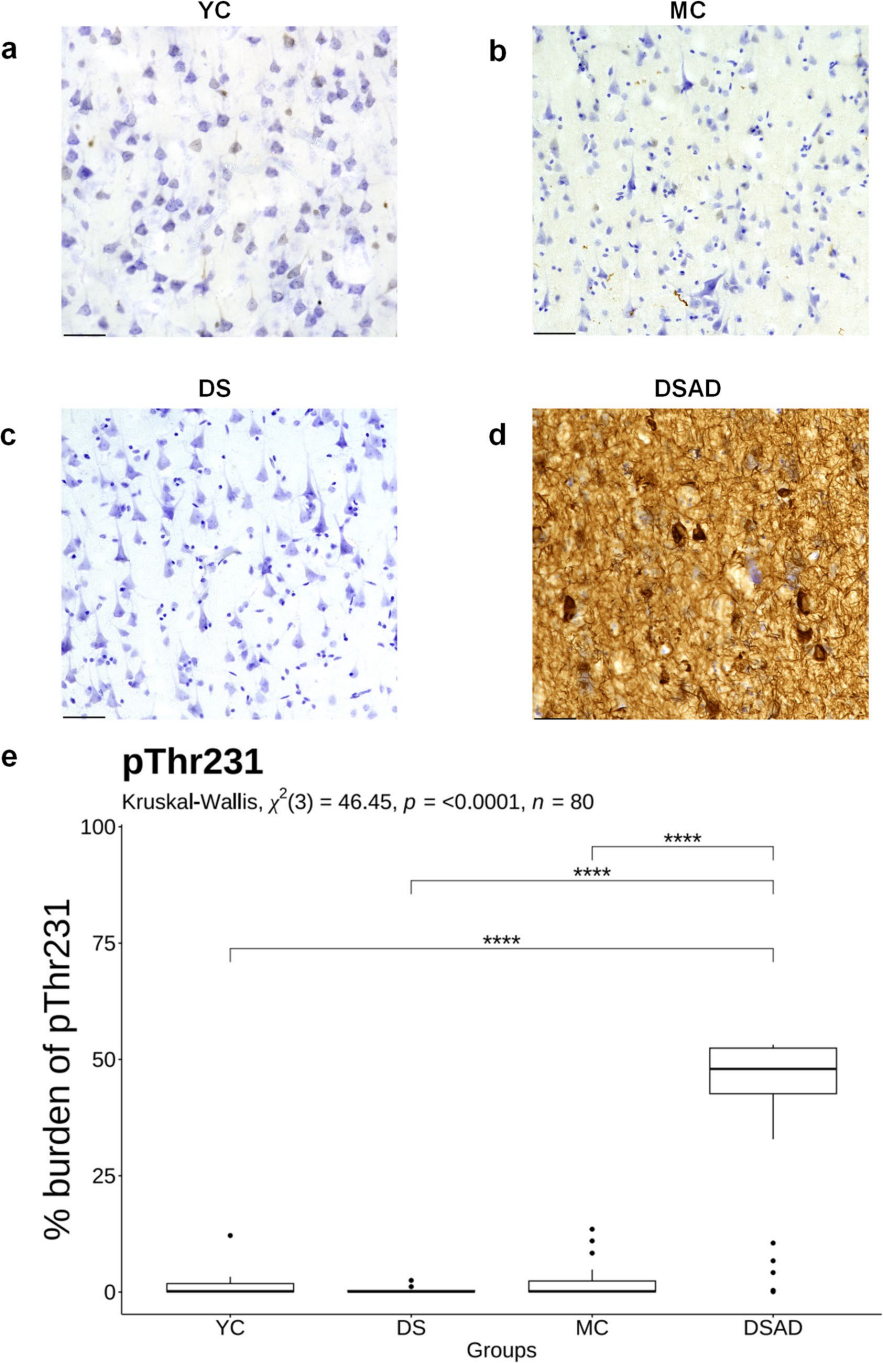
Comparison of pThr231 burden in DSAD and age-matched controls. Representative images of pThr231 immunohistochemical labeling in the frontal cortex of **a** YC, **b** MC, **c** DS, and **d** DSAD. **e** The box plot shows the percentage burden of pThr231 across the groups. Sample sizes per group are as follows: YC (*n* = 15), MC (*n* = 22), DS (*n* = 11), and DSAD (*n* = 32). Statistical comparisons between groups were performed using the Kruskal–Wallis test, followed by Dunn’s post-hoc test with Bonferroni correction. *p < 0.05, **p < 0.01, ***p < 0.001, ****p < 0.0001. *YC* young controls; *MC* middle-aged controls; *DS* young Down syndrome; *DSAD* DS with AD neuropathology; Scale bar = 50 μm

We next tested the hypothesis that all p-tau epitopes would be higher in DSAD relative to DS and age-matched neurotypical controls (YC and MC). There were significant differences in pThr181 (*χ*^2^(3) = 30.94, *p* < 0.0001, *n* = 80) (Fig. 2e), pThr217 (*χ*^2^(3) = 49.73, *p* < 0.0001, *n* = 80) (Fig. 3e), and pThr231 (*χ*^2^(3) = 46.45, *p* < 0.0001, *n* = 80) (Fig. 4e) across the groups. These differences included significantly higher pThr181 burden in DSAD compared to DS (*p* < 0.0001), MC (*p* < 0.001), and YC (*p* < 0.01) groups (Fig. 2). For pThr217, immunohistochemical staining was virtually absent in DS and controls, with significantly higher burden in the DSAD group (DS: *p* < 0.0001, MC: *p* < 0.0001, YC: *p* < 0.0001) (Fig. 3). A similar pattern was seen for pThr231, which showed a significantly higher burden in DSAD relative to all other groups (DS: *p* < 0.0001, MC: *p* < 0.0001, YC: *p* < 0.0001) (Fig. 4). There were no differences in p-tau epitope burden between DS, MC, and YC groups. No sex differences were identified.

### P-tau across the lifespan in DS

**Fig. 5:**
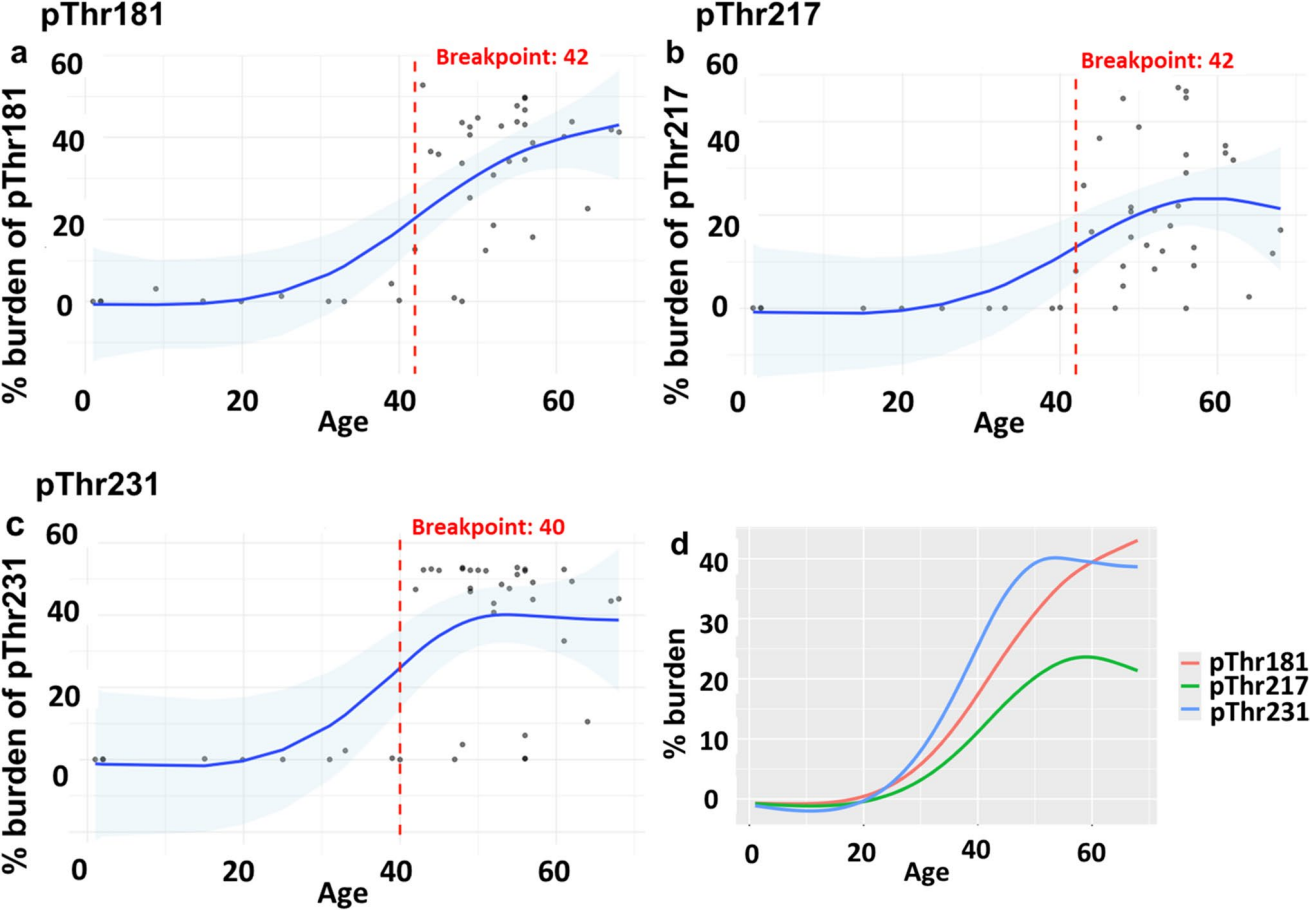
Modeling p-tau epitope burden as a function of age across the DS lifespan. Cross-sectional data from DS and DSAD cases were plotted to examine the age-associated trends in percentage burden of **a** pThr181, **b** pThr217, and **c** pThr231. Models applied: GAM fit with Pettitt estimated breakpoint. Blue line represents the fitted GAM smooths with confidence intervals (shaded blue areas). The vertical red dotted lines represent the estimated breakpoint by the Pettitt test. Dots represent individual DS and DSAD cases (*n* = 43). **d** combined age-associated trends of pThr181, pThr217, and pThr231 burdens. *DS* young Down syndrome; *DSAD* DS with AD neuropathology

We hypothesized that all p-tau epitopes exhibit an age-associated increase, with variation in the age at which they first appear. To identify the approximate age at which each p-tau epitope increases across the DS lifespan in the frontal cortex, GAMs were fitted to the cross-sectional data from DS and DSAD cases (Fig. 5). All three p-tau epitopes showed a steep nonlinear increase in burden with age, clearly delineating the DS and DSAD groups. Moreover, we applied the Pettitt test to estimate a single change point (breakpoint) at which each p-tau epitope burden increased with age. We observed an earlier increasing trend for pThr231, with a breakpoint at age 40 (*p* < 0.01) (Fig. 5c), followed by an estimated breakpoint at age 42 for pThr181 (*p* < 0.001) (Fig. 5a) and pThr217 (*p* < 0.001) (Fig. 5b).

## Discussion

This study characterizes pThr181, pThr217, and pThr231 burdens in the postmortem brains of individuals with DS across the lifespan. Here, we demonstrate that in the frontal cortex, pThr181, pThr217, and pThr231 burdens: (1) are similar in DSAD relative to LOAD; (2) are significantly higher in DSAD compared to DS and controls; and (3) exhibit an age-associated increase across the DS lifespan after 40 years of age.

### P-tau pathology in DSAD and LOAD

We observed similar p-tau epitope burdens in DSAD cases compared to those with LOAD. Although DSAD cases were younger on average, this is in line with emerging evidence suggesting that p-tau pathology develops at an earlier age in DSAD compared to LOAD [63]. Moreover, our findings are consistent with previous neuropathology and biomarker findings [8, 20–22, 38, 40]. For instance, neuropathology observations show that individuals with DS develop p-tau pathology that is virtually identical in distribution and severity to that seen in LOAD [22, 38]. Additionally, the regional progression of p-tau pathology measured by PET in DS is similar to that in LOAD, following the Braak NFT staging pattern [8, 58]. At the fluid biomarker level, increases in CSF total tau and pThr181 in DS are similar to those observed in LOAD [21]. In contrast, our previous study showed higher levels of AT8-positive p-tau pathology in the frontal cortex of DSAD cases compared to LOAD [1], likely due to AT8 recognizing tau phosphorylation at early sites (serine 202 and threonine 205) [37]. Moreover, longitudinal PET imaging in adults with DS revealed that tau pathology emerges within 2.5–5 years following Aβ PET positivity, whereas it emerges within 5 to 10 years in LOAD, indicating a rapid and early progression of tau pathology in DS [63]. Together, these results suggest that while tau pathology development in DSAD reflects that seen in LOAD, its progression may be accelerated, raising important considerations for future therapeutic intervention and responsiveness to amyloid and tau-directed immunotherapies in DS.

### P-tau pathology in DSAD and DS

In this study, we investigated several p-tau epitopes, commonly targeted by biofluid biomarkers in AD, to assess how they differ across diagnostic groups and change with age in the DS brain. Our findings revealed significantly higher burdens of pThr181, pThr217, and pThr231 in the frontal cortex of DSAD cases compared to DS and age-matched controls. This is consistent with our previous study that showed significantly higher levels of AT8-positive p-tau pathology in individuals with DSAD compared to both DS and controls [1]. These findings align with those of prior studies, which have demonstrated that individuals with DS exhibit a significant increase in p-tau pathology across AD staging, both in biofluids (plasma and CSF) and through PET neuroimaging [12, 15, 26, 27, 35]. We observed no significant difference in p-tau epitope burdens between the DS, YC, and MC groups, indicating that p-tau elevation is specific to neuropathologically confirmed AD and supporting previous reports [35, 46]. While fluid biomarkers of pThr181 and pThr217 have been studied in DS, other p-tau epitopes, including pThr231, remain understudied. In non-DS populations, such as LOAD and ADAD, plasma and CSF pThr231 rise at the preclinical stage [3, 43]. Although two subclusters with low and high pThr231 levels were observed in DSAD, the majority of cases showed an increased pThr231 burden.

We observed no sex differences in the p-tau epitopes examined, which agrees with previous findings showing no sex differences in CSF and plasma pThr181 levels among adults with DS [24]. Although plasma and CSF p-tau biomarkers have been studied in the DS population [26, 42], reporting on sex differences remains limited in fluid biomarker studies in DS. In contrast to our findings, an early neuropathology study reported greater neocortical NFT densities in middle-aged females with DS compared to males with DS [52]. Moreover, our previous study also showed a more pronounced association between AT8-positive tau pathology and amyloid plaques in the female occipital cortex relative to the males with DSAD [1]. Although evidence suggests a strong link between female sex and higher tau pathology, we note that the brain regions examined in these previous studies reflect later stages of AD when the occipital cortex is less affected by tau pathology compared to the frontal cortex examined in our study, and the tau outcomes were determined using p-tau variants different from those used in this study.

### Lifespan changes in p-tau in the DS brain

The temporal sequence of fluid and brain p-tau biomarker changes in AD has been studied extensively in the neurotypical population [2, 5, 56], but current evidence is limited with respect to the temporal sequence of p-tau biomarker changes in DS. When modeling p-tau epitope burdens as a function of age across the DS lifespan, we observed that pThr231 burden in frontal cortex increased at age 40 years, followed by increasing pThr181 and pThr217 at age 42 years. Given the overlapping confidence intervals in our GAM fitted data, we hypothesize that increasing p-tau happens within a narrow age window in DS. These age trajectories in p-tau burden closely align with our previous study, demonstrating an initial steep increase in p-tau (AT8) levels in the frontal cortex around the age of 40 [1]. Moreover, our age trajectories of p-tau burden are consistent with prior biomarker studies, which show that fluid (plasma and CSF) pThr181 and pThr217 levels rise by age 40 and are strongly associated with age in DS [14, 20, 21, 35]. Additionally, recent evidence suggests that plasma pThr231 and pThr181 levels begin to rise around the age of 39 years in DS [23]. Our findings also indicate an ordered progression of p-tau variants in the frontal cortex, with pThr231 preceding pThr181 and pThr217. Although evidence for the temporal ordering of p-tau variants in DS is limited, some fluid biomarker studies in AD suggest that pThr231 may increase slightly earlier, followed by pThr217 and pThr181 [2, 3, 36]. Overall, our outcome measures of p-tau pathology through the DS lifespan strongly reflect the increasing trajectories of fluid p-tau biomarkers that become apparent by the fifth decade of life and follow an order sequence similar to that observed across the AD continuum in the neurotypical population [2].

### Limitations

Several limitations warrant consideration. First, our study focused on p-tau epitope burden in the frontal cortex, which limits the ability to infer about p-tau burden vulnerability and progression across multiple brain regions. However, examining the frontal cortex, a key region impacted by AD pathogenesis, across a comprehensive age range provides valuable insights into age-related increase in p-tau variant burdens in DS. Moreover, the use of postmortem cases constrains the ability to infer antemortem events; in vivo tau PET imaging suggests an age-associated rise in tau in individuals with DS [51]. Future studies combining cross-sectional and longitudinal analyses of multiple brain regions are needed to precisely determine the ordinal and temporal sequence in which p-tau epitopes emerge in DS. This may improve characterization of p-tau pathology progression throughout the brain and highlight individual variability. The high individual variability in p-tau epitope burdens, primarily in the DSAD group, could not be explained by systematic contributors, such as age, sex, and PMI; however, other unidentified factors need to be considered. Within and between-subject variations have been reported for plasma p-tau variants in AD and healthy individuals, showing high variability; however, the substantial increase in plasma p-tau observed in AD minimizes variability effects [9]. Although frontal cortical tissue was obtained from three tissue repositories, we applied standardized tissue processing and analysis to minimize possible repository-specific effects on p-tau variant burden measures. Fixation times varied in our cases, and this may have contributed to individual variability in p-tau epitope burdens. Moreover, the agonal state or cause of death may have influenced some of our outcomes; however, it was assumed to represent random error in our groups. Although *APOE* ε4 is known to facilitate tau pathology progression in LOAD [57], our study was underpowered to assess the impact of *APOE* genotype on p-tau burden. Future studies should investigate the effects of *APOE* ε4 on p-tau pathology in these groups. The cross-sectional design of the dataset, inevitable in human postmortem studies, limits the ability to identify the precise age at which p-tau epitope burdens emerge across the DS lifespan. Moreover, the age of regional tau pathology onset remains to be determined in future studies examining multiple brain regions relevant to the Braak NFT staging scheme. Sections from the hippocampus or entorhinal cortex, which would reflect the earliest Braak NFT stages, were not available for most cases included in our study. Although the frontal cortex (BA9) is affected later in AD progression, with Braak stages V/VI [8], the use of this region allowed us to observe progression across the lifespan without reaching maximum burden.

### Conclusions

In conclusion, we demonstrate that brain pThr181, pThr217, and pThr231 burdens are similar between DSAD and LOAD, with higher burdens in DSAD compared to DS and age-matched controls. Furthermore, greater p-tau burdens are associated with advanced age in DS, beginning in the fifth decade of life. Our findings reinforce the similarity in p-tau pathology burden between DSAD and LOAD. Prior biomarker studies linked increasing biofluid p-tau measures to Aβ and p-tau pathology accumulation as measured by PET imaging [26, 35]. The results of those studies are complemented by our current study which focuses on p-tau neuropathology burden in the brain tissue. Additional studies examining the relationship between p-tau epitope burdens and Aβ burden across brain regions are ongoing in our laboratory and will facilitate interpretation of biofluid and neuroimaging biomarker analyses. Collectively, these studies will be valuable to establish the neuropathological correlates of biofluid p-tau assays and p-tau PET imaging biomarkers in DS using cohorts with brain tissue, ante mortem plasma and neuroimaging. Recently, the Roche Elecsys pThr181 plasma test and the Luminpulse G pThr217/B-amyloid 1–42 plasma ratio test received approval from the US Food & Drug Administration (FDA) to aid in the diagnosis of symptomatic AD, in conjunction with other clinical assessments [53, 59]. This establishes a benchmark for comparing other p-tau variants, which may prove instrumental in stratifying populations and identifying disease stages for participant selection and monitoring in AD clinical trials that encompass the DS population [48].

## Supplementary Information

Below is the link to the electronic supplementary material.

Supplementary file1 (DOCX 3862 KB)

Supplementary file2 (XLSX 12 KB)

## Data Availability

Data generated from this study is available from the authors upon request.
