# Supplementary material for: Neuropathological measures of increased tau phosphorylation across the Down syndrome lifespan
Source: Acta Neuropathol. 2026 Mar 22;151(1):28. doi: 10.1007/s00401-026-02994-8 (PMC13006465; doi:10.1007/s00401-026-02994-8)
Supplement: Supplementary file 1 — Supplementary file1 (DOCX 3862 KB) [file 401_2026_2994_MOESM1_ESM.docx]

**Supplementary Information**

**Title:** Neuropathological measures of increased tau phosphorylation across the Down syndrome lifespan

**Journal:** Acta Neuropathologica

**Authors:** Jesse R Pascual^1^, Isabel Rivera^1^, Halyma Nguyen^1^, Phong T Ngo^1^, Alan Hoang^1^, Elizabeth J Andrews^1^, Jeremy Rouanet^1^, Sierra T Wright^1^, Lorena Sordo^1^, Julia Kofler^2,4^, Milos D Ikonomovic^2,3,4,5^, Florence Lai^6^, Mark Mapstone^7^, Bradley T Christian^8^, Benjamin L Handen^9^, Ira T Lott^10^, Eric Doran^10^, Christy L Hom^11^, Jordan Harp^12^, Frederick Schmitt^12,13^, Dana L Tudorascu^9^, Beau M Ances^14^, Michael Phelan^1^, Lei Liu^15^, Lisi Flores-Aguilar^1^, Elizabeth Head*^1^, for the Alzheimer’s Biomarkers Consortium–Down Syndrome (ABC-DS) Investigators

***Corresponding Author:**

Elizabeth Head, MA, PhD.

Department of Pathology and Laboratory Medicine, University of California, 1111 Gillespie Neuroscience Research Facility, Irvine, CA 92697, USA.

Email: [heade@uci.edu](mailto:heade@uci.edu)

**
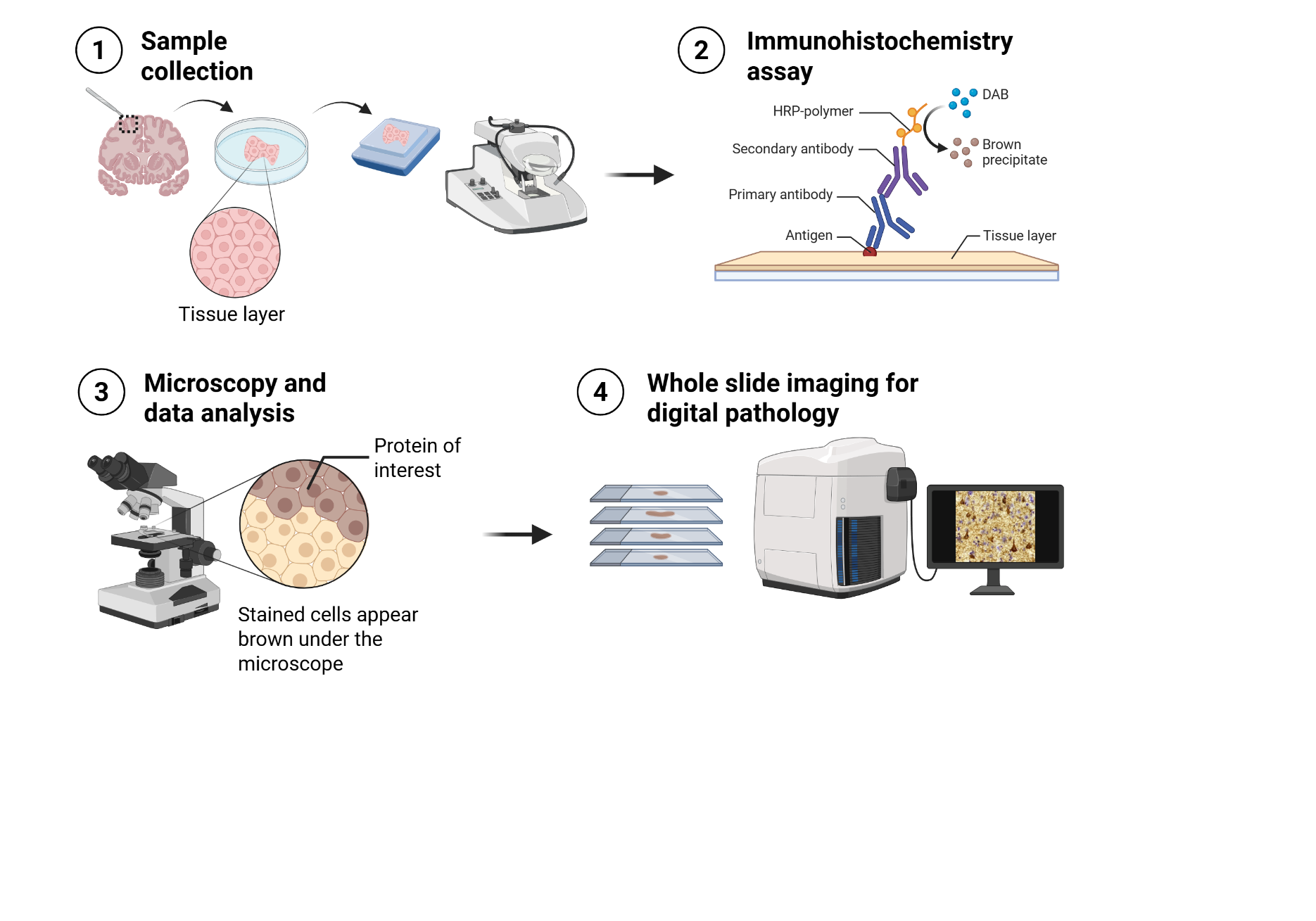
**

**Figure S1** Overview of study design workflow. Study workflow for assessing p-tau epitope burdens in the human postmortem brain. Illustration created in BioRender. Rivera, I. (2025) https://BioRender.com/m040e04

**
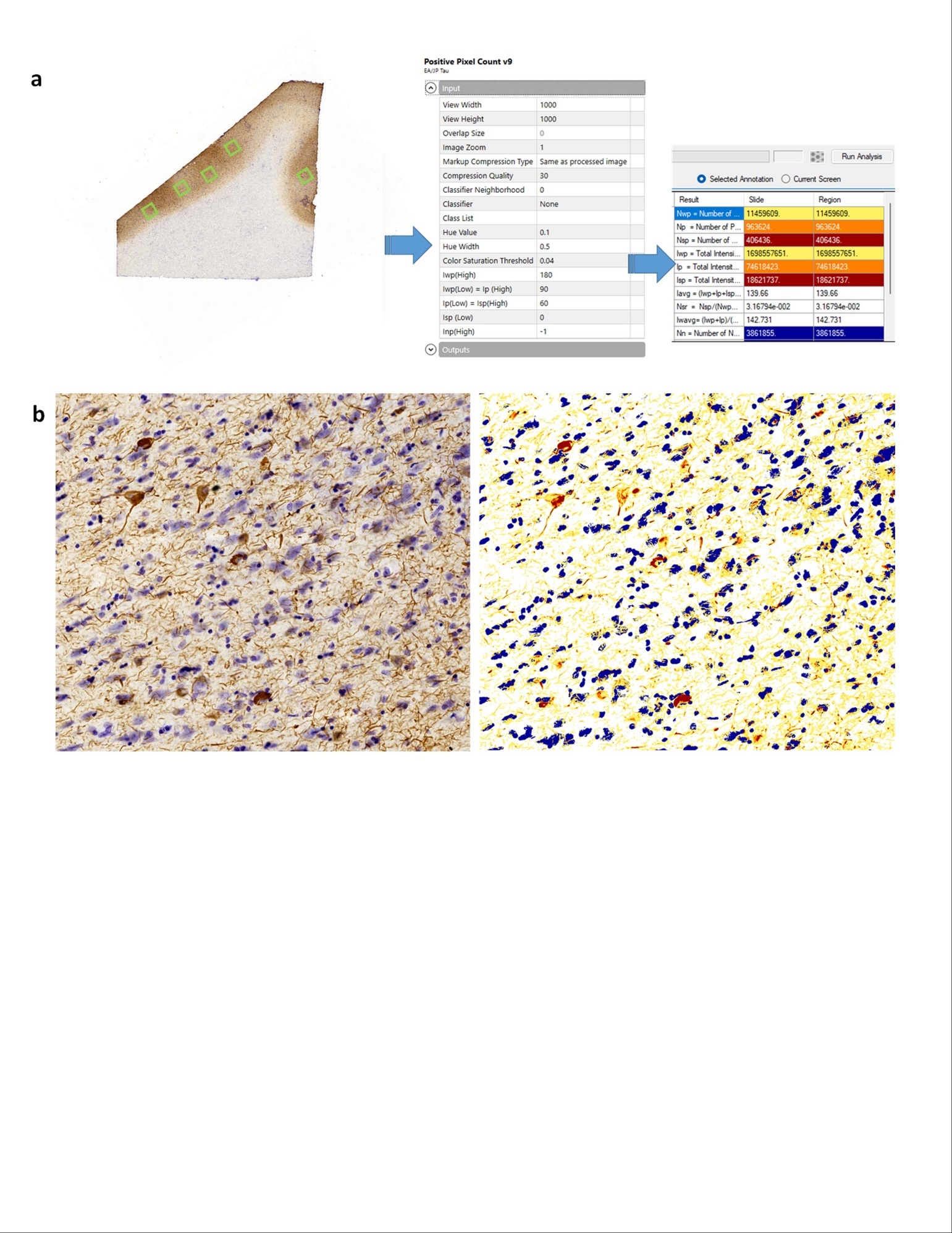
**

**Figure S2** Analysis workflow. (a) The annotation process includes selecting ROIs and detecting positive pixel counts, illustrating input design and output signal positivity in Qupath. (b) The resulting IHC stain (left) and the overlay (right) illustrate how the positive pixel algorithm determines positive p-tau signal (in red/yellow) in relation to the cresyl violet, depicting nuclear staining of cells (in blue), which represents the negative pixels


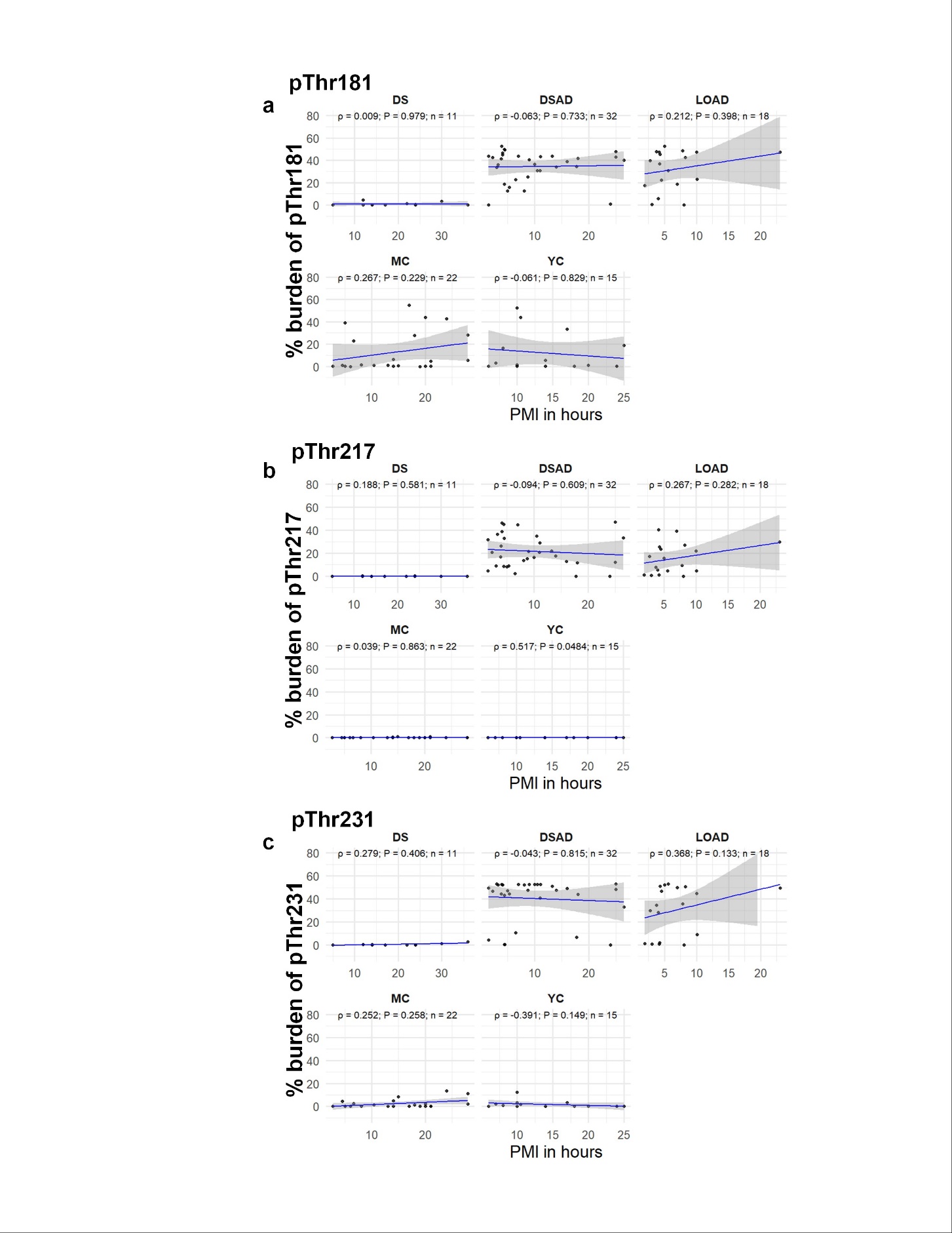


**Figure S3** PMI to p-tau burden outcome measures. Spearman correlation plots for PMI to %burden of (a) pThr181, (b) pThr217, and (c) pThr231. Stratified by diagnostic group. Note: For YC, MC, and DS cases, the % burden of all p-tau values was low and represented noise. YC = young controls; MC = middle age controls; DS = young Down syndrome; DSAD = DS with AD neuropathology; LOAD = Late-onset AD; PMI = postmortem interval


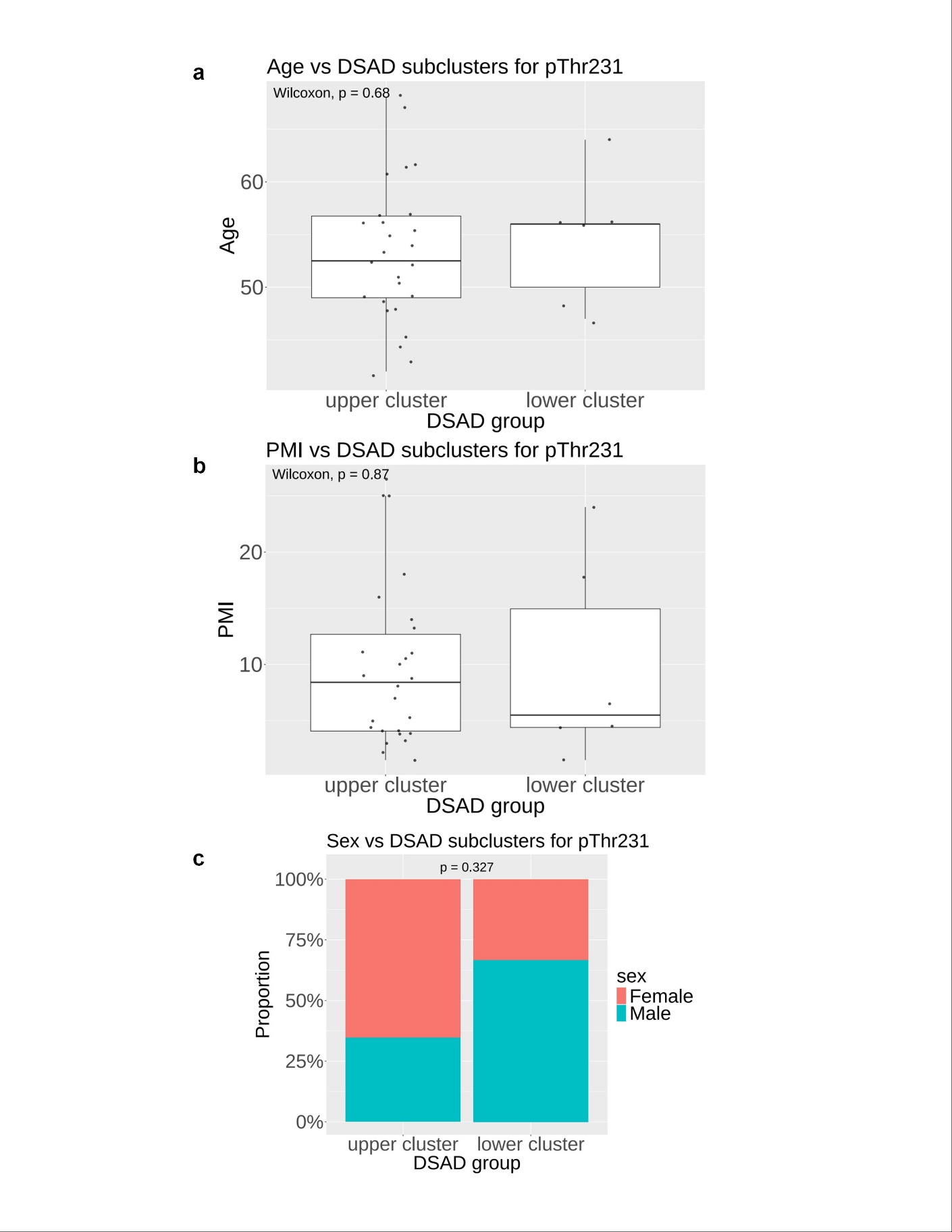


**Figure S4** pThr231 burden in DSAD stratified into two clusters, independent of (a) age, (b) PMI, or (c) sex. Two subclusters identified: high pThr231 burden (upper cluster, n = 26) and low pThr231 burden (lower cluster, n = 6). Statistical comparisons were performed using the Mann-Whitney U (Wilcoxon) test for age and PMI, and a Pearson’s Chi-square test of independence for sex. DSAD = DS with AD neuropathology; PMI = postmortem interval

| **DSAD** | pThr181 | pThr217 | pThr231 |
| --- | --- | --- | --- |
| pThr181 | 1 |  |  |
| pThr217 | .73 (*p* < .001) | 1 |  |
| pThr231 | .38 (*p* = .04) | .49 (*p* = .004) | 1 |
|  | | | |
| **LOAD** | pThr181 | pThr217 | pThr231 |
| pThr181 | 1 |  |  |
| pThr217 | .57 (*p* = .02) | 1 |  |
| pThr231 | .55 (*p* = .02) | .50 (*p* = .04) | 1 |

**Table S1** Associations between pThr181, pThr217, and pThr231 within DSAD and LOAD. Partial Spearman correlations (*r_s_*) are reported with p-values after adjusting for age in DSAD (n = 32) and LOAD (n = 18) groups. DSAD = DS with AD neuropathology. LOAD = Late-onset AD.
